# Word or pseudoword? The lexicality effect in naming and lexical decision tasks during advanced aging

**DOI:** 10.1371/journal.pone.0299266

**Published:** 2024-02-29

**Authors:** Carlos Rojas, Marilyn San Martín, Paula Urzúa, Ernesto Guerra

**Affiliations:** 1 Department of Health Rehabilitation Sciences, Universidad del Bío-Bío, Chillán, Chile; 2 School of Phonoaudiology /Speech Therapy, Universidad de Las Americas, Concepción, Chile; 3 Center for Advanced Research in Education (CIAE), Universidad de Chile, Santiago, Chile; University of Florida, UNITED STATES

## Abstract

Although there is evidence that recognizing pseudowords is more difficult than recognizing words during childhood, adulthood, and early old age (60–75 years), it is not yet clear what happens during advanced aging or the fourth age, a stage when the decline of fluid intelligence strongly affects processing speed, but a good performance of crystallized intelligence is described through an increase in vocabulary and knowledge. The objective of this study was to determine the lexicality effect in advanced aging, specifically exploring how the ability to recognize words and pseudowords (ortho-phonologically plausible for Spanish) is affected during the third and fourth-ages. The lexicality effect was measured using naming and lexical decision tasks. Response time and accuracy were compared between a fourth-age group (80+ years) and two third-age groups (60–69 and 70–79 years) through linear regression models. The results showed that, in general, the fourth-age group had longer response times and reduced accuracy when recognizing words and pseudowords. Moreover, they showed a significant lexicality effect (which increases from the third- age onwards), reflected in higher costs during pseudoword recognition, especially when the task required more cognitive effort (lexical decision task). These results were consistent with the impact of the deterioration of fluid intelligence on the speed of lexical recognition and with the better performance that crystallized intelligence can generate on accuracy, especially in the early stages of old age. Additionally, this study supports the fact that pseudoword recognition resists cognitive decline, as accentuated deterioration is visualized only after 80 years.

## Introduction

People in advanced aging, also known as the fourth-age (80 years and older [1]), have had various experiences that differ depending on demographic, historical, psychological, and socioeconomic factors [2, 3]. Therefore, cognitive performance data in advanced aging show a high dispersion, with significant individual differences [4]. However, several studies [4–7] report that fourth-age elders, like those in the third-age (60 to 79 years), retain certain skills (i.e., vocabulary and knowledge) while decline progressively others (i.e., operational capacities) until they reach a minimum level of functioning, leading to a generalized cognitive decline before death [4]. Furthermore, perceptual deficits become more pronounced [7, 8] and processing speed continues to decline [5, 9] during the fourth-age. Nevertheless, there is still little knowledge about the critical points that characterize the cognitive and linguistic evolution of people with advanced aging. Therefore, more evidence is needed to determine what is altered and at what level [10, 11].

But what do we mean when we talk about older people losing some cognitive abilities and maintaining or improving others? For Cattell [12] the construct of intelligence is divided into two types: fluid and crystallized. The former is essentially the ability to deftly solve problems in novel situations, while the latter represents knowledge and skills acquired through experience. During aging, the decline in fluid intelligence is reflected in a decrease in the ability to think logically and solve problems in unfamiliar situations. It also includes a decline in abstract reasoning and adaptation to new situations. This type of intelligence is based on innate biological abilities, is less susceptible to the influence of education and culture [5–7], and peaks in adolescence. Crystallized intelligence, on the other hand, tends to increase with age and is reflected in the accumulation (and use) of knowledge acquired throughout life [4–6, 9]. It is highly dependent on education, cultural exposure, and life experiences, and remains relatively stable over time. It is also associated with the ability of older people to use knowledge and experience to solve specific problems and tasks [4–7, 9].

On the other hand, although language processing seems to endure the passing of the years, there is evidence that language deficits may appear from the age of 60 onwards, increasing from 70 onwards [13], dominated by a strong impairment in production versus a relative maintenance of comprehension [14, 15]. At the lexical level, older people stand out for a visible slowdown in word recognition [7], accentuated from the age of 80 and onwards, with an increase in the error rate during a cognitively demanding task [16]. Additionally, word recognition tasks show that the behavior -of facilitation or inhibition- exhibited by certain variables such as lexical frequency, imaginability and positional syllable frequency remain stable throughout the life cycle, fourth-age included [16]; nevertheless, there is no certainty about what happens with the lexicality variable in advanced aging, i.e., when a person aged 80 years and older must visually distinguish between a set of meaningless letters compared to one that does have meaning.

Several studies have shown that during childhood, adulthood, and early old age (60–75 years), recognizing and reading a sequence of letters that has no meaning (pseudoword) can be more challenging than recognizing "real" words [17, 18]. A plausible explanation for this phenomenon is that pseudowords require greater cognitive effort due to the fruitless search that generates a non-existent representation in the mental lexicon. Conversely, words can access the lexicon quickly, accurately, and efficiently [19, 20]. Therefore, studying how the lexicality variable behaves in advanced stages of old age would be intriguing, given the greater cognitive effort required to recognize pseudowords. This is particularly relevant when the decline of fluid intelligence affects the speed of information processing [4, 5, 21], but crystallized intelligence could potentially enhance response accuracy through the adequate performance of vocabulary, experience, and knowledge [22, 23].

Empirically speaking, tasks involving lexical recognition—both in young and elderly individuals—with low cognitive loads such as naming tasks (which only require reading a sequence of letters, [17, 24]), or experiments requiring a decisional factor such as the lexical decision task (LDT), show that pseudowords produce higher response times (RT) and lower accuracy than word recognition [25, 26]. However, such differences do not necessarily reach significance levels. It has been suggested that the higher recognition cost generated by pseudowords (slower and less accurate) could be explained by the verification work performed by the individual to ensure that the set of letters presented does not correspond to a word. In contrast, rapid access in word recognition could be explained by the "saving mode" related to speed and cognitive resources associated with the use of the lexical reading pathway [27], which allows for almost simultaneous access to the lexicon and then to the meaning of the word [19, 28].

New questions arise about how the lexical word-processing pathway behaves in the fourth-age, when cognitive decline is generalized. For example: to what extent do pseudoword recognition difficulties increase after the age of 80? Are there significant differences between word vs. pseudoword recognition in TR and accuracy at this age? Finally, are there significant differences in word and pseudoword recognition between the third and fourth-age?

It should be noted that not all pseudowords are equally difficult to recognize. The cost of processing will depend largely on their level of similarity (ortho-phonological) to a word [17, 24, 29]. Pseudowords with "real" words as lexical neighbors and orthographic and phonological structures plausible for the tested language will take longer to be rejected, causing more interference in the recognition system compared to pseudowords with no similarity to any word. In this case, it is hypothesized that a person carries out an intense search in the visual lexical module for the corresponding lexical item, which ends up being unsuccessful [17, 24].

The response intention presented by the person when locating a lexical portion within the pseudoword (ortho-phonological similarity) may momentarily induce them to give an affirmative response that must be quickly corrected. On the other hand, a pseudoword lacking phonological neighbors and orthographic and phonological rules plausible with the tested language will be quickly recognized as such. This will result in lower RT and fewer errors, as there is no interference of "similarities" in the recognition system [17, 24, 29]. Hence, it is interesting to explore the effects of lexicality throughout aging when processing plausible pseudowords for the speaker’s native language, generating more cognitive load.

Moreover, the recognition of pseudowords may depend on the number of letters they contain. For instance, there is evidence of a direct association between pseudoword length and phonotactic probability (defined as the probability in which a sequence of sounds occurs in a language [30]). Longer pseudowords would increase phonotactic probability, while shorter ones would reduce it [31]. However, Vitevitch & Luce [32] demonstrated a strong correlation between phonotactic probability and phonological neighborhood density. Words with high neighborhood density, regardless of their length, tend to have high phonotactic probability.

Del Pino et al. [33] showed, using psychometric tasks, that the detection of pseudowords begins to deteriorate significantly in the range of 71 to 75 years and older, rather than earlier stages. They concluded that the recognition of pseudowords would only be impaired in advanced stages of old age and, therefore, this type of linguistic stimuli would be more resistant to cognitive decline due to aging. Similarly, two decades earlier, Patterson et al. [34] had already stated that reading pseudowords would estimate premorbid cognitive performance more accurately than reading low-frequency words. Reifegerste et al. [35] also investigated the effects of lexicality in healthy young and older adults (all bilingual and of the third-age) using LDT. The authors observed no interaction between age and pseudoword recognition, concluding that lexicality would not be affected in early stages of aging.

Despite the large amount of accumulated evidence on word and pseudoword processing in school populations with learning disabilities, language, and bilingualism, studies linking the cognitive effects of aging, particularly advanced aging, on lexicality and pseudoword recognition are almost non-existent [33]. Therefore, beyond the scarce studies on the topic, available evidence suggests that studying the effect of lexicality in advanced aging would not only determine the capacity for lexical recognition (which is essential for proper language comprehension) at this stage of the life cycle but also provide insights into whether pseudoword processing is resistant to the passing of years and normal cognitive decline.

Furthermore, studying the lexicality effect in old age is cognitively relevant for several reasons: first, it provides insight into how cognitive changes related to very advanced aging such as the reduction of fluid intelligence (processing speed, problem-solving, decision making and inhibition, among others) or the maintenance of crystallized intelligence (vocabulary and expertise) may influence the ability of older people to recognize words and pseudowords. In addition, understanding limitations in recognizing pseudowords during aging could help to identify possible difficulties in reading (visual recognition) and language comprehension (auditory recognition), which could affect quality of life and social interaction [33]. Moreover, the study of the lexicality effect during aging may also contribute to a better understanding of language processing impairments associated with neurodegenerative diseases (Alzheimer’s disease or other dementias), as it could help characterize these deficits, contribute to early diagnosis, and identify possible cognitive markers [35].

### The present study

This study examines the impact of lexicality on advanced aging, specifically investigating how the ability to recognize words and ortho-phonologically plausible pseudowords in Spanish is affected during the third and fourth-ages. Exploring the effect of lexicality in old age is important because it sheds light on how cognitive changes related to advanced aging (fluid/crystallized intelligence) may affect the ability of these individuals to recognize words and pseudowords, which could lead to difficulties in reading or listening comprehension. Two behavioral visual recognition experiments are conducted to assess the effects of aging on reaction time (RT) and accuracy. The first involves a naming task, examining how word and pseudoword recognition is affected when older people only have to read the presented stimulus [36]. The second method involves a cognitive decisional factor through LDT, which examines how word and pseudoword recognition behaves when the person has to decide whether or not the presented stimulus is a word of their language [37]. It is hypothesized that the fourth-age group will be slower in recognizing both words and pseudowords in both tasks compared to their younger peers due to generalized cognitive deficits inherent in later stages of aging, associated with deficits in fluid intelligence such as reduced processing speed, problem solving, decision-making, and inhibition. This may impair overall comprehension during advanced aging. Simultaneously, a lexicality effect is expected in both the third and fourth-age groups, which results in higher RT in the recognition of pseudowords than words, due to the greater cognitive effort generated by the unsuccessful search for pseudowords. Nevertheless, both groups would exhibit a correct accuracy rate in distinguishing words and pseudowords, as a result of appropriate conceptual performance (crystallized intelligence) observed during the different phases of normal aging. Finally, both the third and fourth-age groups are projected to have higher pseudoword processing costs in LDT given the cognitive decisional factor involved in this task.

## Materials and methods

### Experiment 1

The naming task is a recognition method that involves participants reading aloud each presented stimulus, without making a decision. This approach activates lexical cognitive mechanisms and eliminates the cognitive decisional effect of deciding whether the presented set of letters is a word or not. As a result, it reduces categorical post-lexical effects, such as delayed post-recognition semantic activation, that could increase the complexity of the task by increasing cognitive demand. The task assumes that the time required to read aloud the stimulus is determined by the recognition of lexical representations corresponding to the sensory input. This process imposes low additional cognitive load, which can facilitate recognition at very late stages of aging.

#### Participants

Ninety older adults participated voluntarily in this study. They were divided into three groups of 30 individuals each according to their age. Group third-age 1 included individuals aged 60–69 years (12 men, 18 women; average age = 65.7 years, SD = 2.99; average years of education = 13, SD = 1.23); group third-age 2 included individuals aged 70–79 years (14 men, 16 women; average age = 74.0 years, SD = 2.89; average years of education = 13.1, SD = 1.81); and group fourth-age included individuals aged 80–92 years (8 men, 22 women; average age = 82.5 years, SD = 3.10; average years of education = 13.03, SD = 1.71).

All older adults belonged to three local senior clubs associated with the university. To participate, they had to meet the following inclusion criteria: be 60 years of age or older, have completed at least 8 years of schooling, self-report as actively aging (mentally, physically, and socially), have normal (or corrected) hearing and vision, have urban residence, and complete the experiments within a maximum period of 2 months. Exclusion criteria included having a history of cerebrovascular disease, a diagnosis of neurodegenerative disease, depression or a psychiatric illness, and risk scores on any of the following tests applied: Montreal Cognitive Assessment (MoCA score of < 21 points [38]), GSD-15 (score of > 11 points [39, 40]), or Boston Reading Comprehension subtest score of < 4 points [41]. In addition, participants’ medical care records had to be up-to-date, indicating optimal cognitive performance. Approximately 140 older individuals were invited to participate. Of those interested in participating, those who did not meet the inclusion and/or exclusion criteria were excluded. All participants declared that they were monolingual.

To participate, all older persons read and signed (in writing) an informed consent form approved by the Ethics Committee of the Universidad de Concepción, Chile (ID: 21170718). One signed copy remained in the participant’s possession, and another signed copy remained in the possession of the investigator in charge of the project. The objectives and details of the study were presented to the authorities of each club. Then, older adults interested in participating were assessed for cognitive (MoCA), emotional (GSD-15), and basic reading comprehension (Boston) performance. Finally, the selected older individuals were invited to the University’s Specialty Laboratory to perform the naming task and LDT. The recruitment period of the participants took place between November 21, 2019, and May 20, 2020.

#### Materials and design

The experiment consisted of a total of 150 trials. It included 60 words, 30 high-frequency nouns/adjectives extracted from the Spanish Lexical Database (EsPal, https://www.bcbl.eu/databases/espal/). EsPal is a database designed to accommodate the diversity of the Spanish language (Spanish and Latin American) at the phonological and lexical levels [42]. For example, allows the user to choose which phonological representation to use to derive properties related to the phonological neighborhood of words (Spanish or Latin American). Moreover, at the lexical frequency level, it allows the selection of words based on the specific parameters (in this study the following parameters were used: high frequency = absolute frequency equal to or higher than 14.0 [number of occurrences of the word in the EsPal corpus divided by the total number of words in the corpus multiplied by one million], range 418.5 [day] - 14.03 [shoes], average value 96.56; low frequency = absolute frequency between 0.60 and 1.57, range 0.61 [clone] - 1.57 [champagne], average value 0.95). Thus, the selected words were phonologically adapted to Latin American Spanish from a set of more than 1000 items. At the same time, the researchers ensured that each of the selected words represented high and low-frequency words for Latin American Spanish.

The selected words were counterbalanced subdivided into high (30) and low (30) positional syllable frequency of the first syllable, categorized according to the dictionary by Álvarez et al. [43]. In addition, all words presented a high number of phonological neighborhood (density higher than 5.0, according to the Spanish Lexical Database). Finally, the experiment included 60 ortho-phonologically plausible pseudowords for Spanish (considered difficult to recognize as they generate greater cognitive effort and interference during old age). All pseudowords had the same syllabic structure as words, were bisyllabic, and had high density of phonological neighbors and high phonotactic probability according to the criteria of Storkel [31] and Vitevitch & Luce [32]. The experiment also included 30 filler trials. 15 fillers consisted of words classified as medium lexical frequency and medium PSF (to provide a more ecologically valid range of word frequencies/PSF and to minimize potential strategic artifacts). In addition, 15 pseudowords, similar in syllable structure to filler words and ortho-phonologically plausible for Spanish, were presented. Finally, the experiment also included 5 training trials (see S1 File. List of trials by experimental task).

#### Procedure

The participants were seated in an individual, lighted, and acoustically isolated room. Trials were presented on a 15.6-inch computer screen using E-Prime3.0 software. The task was administered in two rounds. It began with instructions and 5 training trials. Later, words and pseudowords were randomly delivered. Each trial started with a warning sign in the center of the screen for 1000ms, immediately after delivering the target written in capital letters. For the spoken response to be recorded, an "InLine" 2300 ms was added. InLine is an optional tool (or object) of the E-Prime software, preferably used to capture all types of responses to a target. It is also useful in situations where standard software objects do not provide the flexibility required by certain paradigms. In the experiments carried out, the InLine tool facilitated the integral capture and recording of participants’ oral responses.

To finish, feedback to the response was given with a 1000 ms "response registered" prompt; then, the next trial began. In the event of no response after 10 seconds, the examiner pressed key 1 on the keyboard, recording the response (not counted as valid data) and allowing the next trial to proceed. The task consisted of participants naming aloud each of the words and pseudowords presented on the screen. They were instructed to respond quickly and without error when the stimulus appeared. The computer, using the Chronos voice key, monitored the time elapsed from the presentation of the stimulus until the participant responded orally, as well as recording right guesses and errors.

#### Data analysis

First, a manual analysis of each trial was carried out to identify correct or incorrect responses (a response was only considered correct when the expected visual stimulus was named exactly). The errors consisted mainly of misreading words (in a few cases) and pseudowords. In addition, some errors were observed in the regularization (or lexicalization) of pseudowords, i.e. several pseudowords were read as "real" words. Then, the reaction time (RT) of each trial was checked to exclude trials with outliers. Trials with very long (>6,000 ms) or very short (<200 ms) latencies were eliminated from the final analysis (invalid data), similar to the criterion of Ratcliff et al. [44, 45]. Additionally, trials having a response by-product of involuntary activation of the vocal cue were not considered valid (i.e., activations resulting from an unexpected verbal comment, a strong exhalation, hesitating, coughing, and rubbing against the microphone). Invalid trials were equivalent to 2.28% of the experiment. Prior to the inferential analysis, the RT data were logarithmically transformed to fit a normal distribution, and the accuracy data were only fitted to a binomial distribution. The statistical analysis was performed using regression models with cross-mixed effects. The lme4 [46] and lmerTest [47] packages of the R Project statistical software were used, allowing intrinsic variability accommodation at the participant and item level in a single regression without needing to add more data. Once the descriptive statistics of the data (M and SD) were calculated and their distribution adjusted, linear models were run for the analysis of the RT variable and generalized linear models for accuracy. As for the analysis, the models included as predictors: age group (60-69/ 70-79/ 80–92 years old) and lexicality (word/pseudoword). All models incorporated interactions between variables and included random intercepts at the participant and item level as well as random slopes for the experimental variables. Due to the research interest in the fourth age group, the overall average of this group served as the regression intercept, comparing this group directly to the third-age groups and the lexicality factor. To make the decision to divide the participants into 2 groups of third age and 1 group of fourth age, we relied on the study of Verhaegen & Poncelet [13], who found that lexical-semantic difficulties appear from the beginning of aging (60 years) and begin to accentuate from the age of 70. On the other hand, to define the 80+ group, we rely on the studies of Höpflinger [1], Mitchell et al. [5], and Poon et al. [9], who found that a marked cognitive decline can be observed from this stage onward. Therefore, this evidence and our research objective encouraged us to create these 3 "aging groups". Finally, we also conducted post-hoc test pairwise comparison (Bonferroni corrected) to assess the lexicality effect on each third-age groups, using the same multilevel regression approach. The models run for the statistical analysis of Experiment 1 were as follows: RT = Imer(logRT ~ (group60-69 + group70-79) * Lexicality + (1 + Lexicality | Participant) + (1 + group60-69 + group70-79 | Item)). Accuracy = glmer(acc ~ (group60-69 + group70-79) * Lexicality + (1 + Lexicality | Participant) + (1 + group60-69 + group70-79 | Item)).

In addition, and to complement the analysis proposed for Experiment 1, two alternative linear regressions (for RT and accuracy) were designed using age as a continuous variable. Further details can be found in Tables A1 and A2 and Figs A1 and A2 in the S2 File.

## Results experiment 1

**Fig 1:**
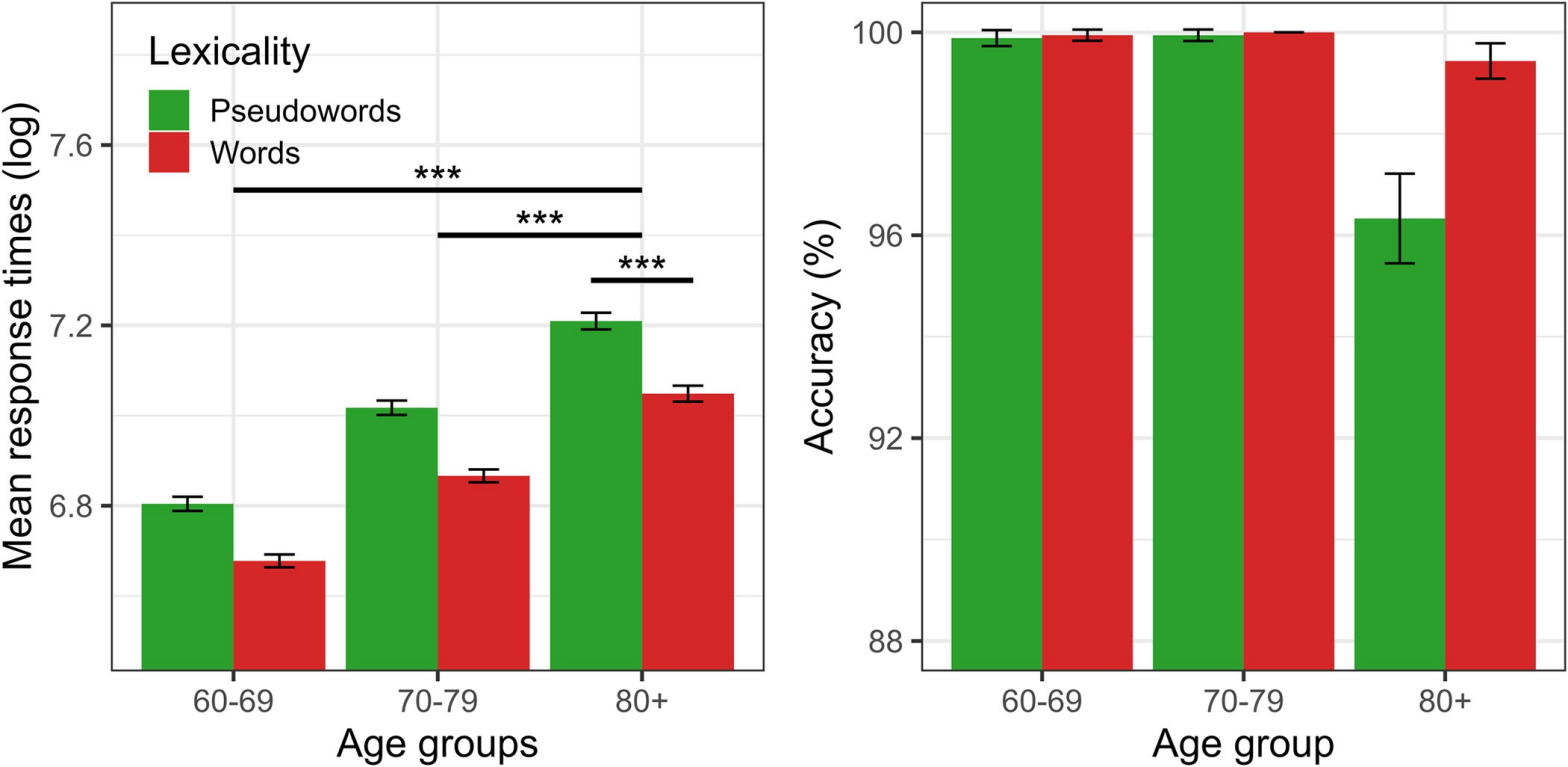
Mean log-transformed RT (left panel) and accuracy percentage (right panel) as a function of age range (80+, vs. 60–69 and 70–79) and lexicality (words vs. pseudowords) in the naming task for experiment 1. Horizontal black lines present contrasts and the stars show significance level (*** = p < .001; ** = p < .01; * = p < .05). Error bars represent within-subject adjusted 95% confidence intervals.

Linear regression analysis on participants’ RT (Table 1) shows that the fourth-age group was significantly slower recognizing words and pseudowords compared to both third-age groups (group 60–69: β = -0.186, se = 0.021, t = -8.763, p<0.00; group 70–79: β = -0.091, se = 0.021, t = -4.319, p<0.00). Moreover, the regression model shows that the fourth-age group exhibited a main effect of lexicality (β = -0.074, se = 0.010, t = -7.231, p<0.001), where words obtain a faster RT (lower recognition cost) than pseudowords. Furthermore, no significant interaction effects were observed between both third-age groups and lexicality. Finally, post-hoc test pairwise (Bonferroni corrected) showed a reliable effect of lexicality on participants RT, both in the young third-age group (group 60–69: β = -0.064, se = 0.009, t = -6.462, p<0.001) and the older third-age group (group 70–79: β = -0. 07584, se = 0.010, t = -7.209, p<0.001), reflecting faster answers (recognition facilitation) for words over pseudowords. However, the differences between words and pseudowords were smaller in these groups than shown by the fourth-age group, reflecting that the lexicality effect could increase with age (see Fig 1, left panel).

**Table 1:** Linear mixed-effects regression response time (log) results for naming task.

Response Time (RT)	Estimate	se	t	Pr(>|t|)	
*Intercept (fourth-age)*	6,940	0,021	335,335	0,000	***
Group 60–69 (words + pseudowords)	-0,186	0,021	-8,763	0,000	***
Group 70–79 (words + pseudowords)	-0,091	0,021	-4,319	0,000	***
Lexicality (words vs. pseudowords)	-0,074	0,010	-7,231	0,000	***
Group 60–69: Lexicality (words vs. pseudowords)	0,009	0,004	1,931	0,056	
Group 70–79: Lexicality (words vs. pseudowords)	0,003	0,004	0,672	0,503	

*** = p < .001

** = p < .01

* = p < .05

On the other hand, in the generalized linear regression on accuracy (Table 2) no significant differences were obtained in the overall accuracy rate (in both word and pseudoword recognition) between the fourth-age group compared to both third-age groups. At the lexicality level (words versus pseudowords), no significant differences were observed either in the fourth-age group or in any of the interactions with the third-age groups, which can be explained by the ceiling effect produced by the high level of accuracy (over 96%) in the recognition of both types of stimuli throughout aging (see Fig 1, right panel), and also because the differences in accuracy between words and pseudowords were at a small-scale (less than 3% in the fourth-age group, and less than 1% in both third-age groups). Post-hoc test pairwise comparison (Bonferroni corrected) shows no reliable effect of lexicality on accuracy in the third-age groups (all p-values > 0.025).

**Table 2:** Generalized linear mixed-effects regression accuracy results for naming task.

Accuracy	Estimate	se	z	Pr(>|z|)
*Intercept (fourth-age)*	15.851	399.950	0.040	0.968
Group 60–69 (words + pseudowords)	3.470	2.188	1.586	0.113
Group 70–79 (words + pseudowords)	5.711	568.666	0.010	0.992
Lexicality (words vs. pseudowords)	1.630	397.247	0.004	0.997
Group 60–69: Lexicality (words vs. pseudowords)	-0.578	1.746	-0.331	0.741
Group 70–79: Lexicality (words vs. pseudowords)	1.855	564.824	0.003	0.997

*** = p < .001; ** = p < .01; * = p < .05

### Experiment 2

In the lexical decision task (LDT), participants are asked to quickly determine whether a presented stimulus is a word in their language or not. To perform this task, the participant consults the visual lexical module to see if the sequence of processed letters matches a lexical representation stored in their mental lexicon. The response time (RT) provides an indication of the cognitive resources used in recognizing the stimulus. The assumption of the LDT is that the time required for the participant to respond to the task and make a decision consistent with the presented stimuli is determined by the recognition of the representations corresponding to the sensory input, which will be influenced by the cognitive load of the task, and which may increase RT and reduce accuracy during old age.

#### Participants

The sample that participated in experiment 1 also took part in experiment 2.

#### Materials and design

The experiment presented a total of 150 trials (in addition to those used in the naming task). It contained 60 words, 30 high-frequency nouns/adjectives (absolute frequency equal to or higher than 14.0) and 30 low-frequency words (absolute frequency between 0.60 and 1.0), extracted from the Spanish Lexical Database (https://www.bcbl.eu/databases/espal/). The same words were counterbalanced and subdivided into high (30) and low (30) imaginability, categorized according to a pilot study. In addition, all words presented a high number of phonological neighbors (density higher than 5.0, according to the Spanish Lexical Database). Finally, the experiment contained 60 ortho-phonologically plausible pseudowords for Spanish (which are considered difficult to recognize as they generate greater cognitive effort and interference during old age). All pseudowords were of similar syllabic structure as words, and were bisyllabic and trisyllabic, with high density of phonological neighbors and high phonotactic probability according to the criteria of Storkel [31] and Vitevitch & Luce [32]. The experiment also included 30 filler trials (also required yes/no answers). 15 fillers consisted of words classified as medium lexical frequency and classified as medium imaginability according to normative (to provide a more ecologically valid range of word frequencies/imaginability). In addition, 15 pseudowords, similar in syllable structure to filler words and ortho-phonologically plausible for Spanish, were presented. Finally, the experiment also included 5 training trials (see S1 File. List of trials by experimental task).

#### Procedure

The LDT procedure was similar to experiment 1, with the same structure in each trial and number of stimuli, and was administered in two blocks. However, unlike experiment 1, this task required participants to decide whether the presented stimulus was a word or not, by means of the oral response "yes" for words and "no" for pseudowords.

#### Data analysis

The manual analysis of each trial followed the same criteria as for experiment 1. Eliminated or invalid trials accounted for 4.42% of the total experimental data. Before statistical analysis, the RT data were transformed to logarithmic function and fitted to a normal distribution, while the accuracy data were fitted to a binomial distribution. For inferential analysis, mixed linear regression models from the lme4 [46] and lmerTest [47] packages of the R Project statistical software were used. Linear models were used for RT analysis and generalized models for accuracy. The models included age group and lexicality as predictors. All models incorporated interactions between variables and included random intercepts at the participant and item levels. As in Experiment 1, and justified on the same theoretical and technical grounds, the fourth-age group was used as the intercept, comparing this group directly with the third-age groups and the lexicality factor. In addition, post-hoc test pairwise comparisons (Bonferroni corrected) were conducted to assess the lexicality effect on each third-age group, using the same multilevel regression approach. The models run for the statistical analysis of Experiment 2 were as follows: RT = Imer(logRT ~ (group60-69 + group70-79) * Lexicality + (1 + Lexicality | Participant) + (1 + group60-69 + group70-79 | Item)). Accuracy = glmer(acc ~ (group60-69 + group70-79) * Lexicality + (1 + Lexicality | Participant) + (1 + group60-69 + group70-79 | Item)).

Finally, and to complement the analysis proposed for Experiment 2, two alternative linear regressions (for RT and accuracy) were designed using age as a continuous variable. Further details can be found in Tables A3 and A4, Figs A3 and A4 in the S2 File.

## Results experiment 2

**Fig 2:**
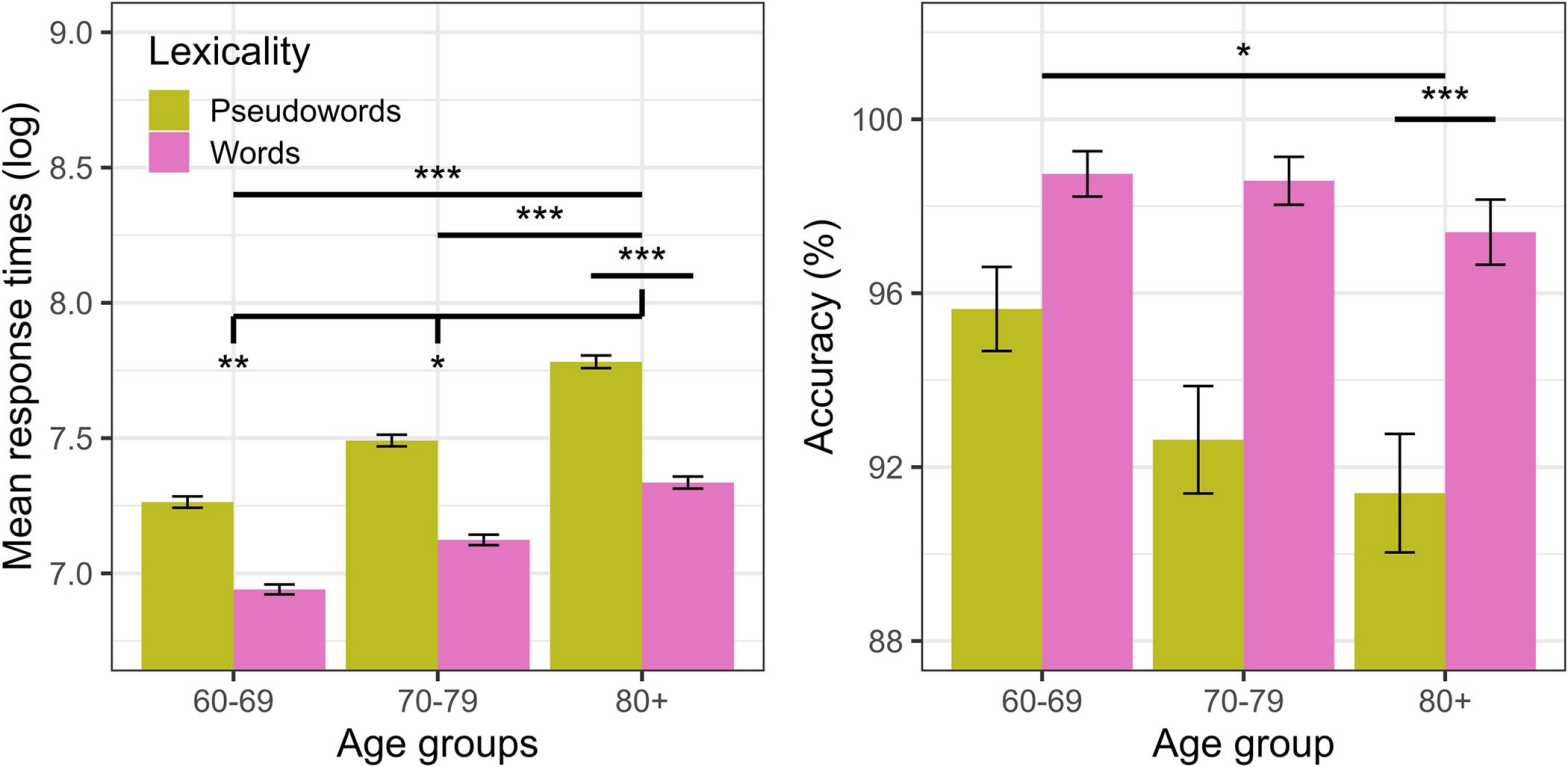
Mean log-transformed RT (left panel) and accuracy percentage (right panel) as a function of age range (80+, vs. 60–69 and 70–79) and lexicality (words vs. pseudowords) in the naming task for experiment 2. Horizontal black lines present contrasts and the horizontal lines with vertical dash represent the interactions. Stars show significance level (*** = p < .001; ** = p < .01; * = p < .05). Error bars represent within-subject adjusted 95% confidence intervals.

The linear regression mixed-effects on participants’ RT (Table 3) displays a main effect of fourth-age compared to both third-age groups, where the latter responded significantly faster for both words and pseudowords (group 60–69: β = -0.225, se = 0.020, t = 88.340, p<0.00; group 70–79: β = -0.125, se = 0.020, t = 87.858, p<0.00). Likewise, the fourth-age showed a main effect on lexicality, with faster responses (recognition facilitation) for words over pseudowords (β = -0.193, se = 0.016, t = 173.364, p<0.00). Additionally, significant interaction effects were observed between groups 60–69 (β = 0.031, se = 0.010, t = 91.930, p = 0.002) and 70–79 (β = 0.020, se = 0.009, t = 89.896, p = 0. 041) and lexicality. These interactions reflect an increase in the difference between RT for words and pseudowords with an increase in age, suggesting that the older the participant, the greater the difference in RT between words and pseudowords (or increased lexical effect; see Fig 2, left panel). In addition, the difference between words and pseudowords is also significant in both third-age groups (age 60–69, β = -0.164, se = 0.017, t = -9.366, p<0.001; age 70–79, β = -0. 187, se = 0.018, t = -10.360, p<0.001), as reflected in the post-hoc test pairwise analysis (Bonferroni corrected).

**Table 3:** Linear mixed-effects regression response time (log) results for LDT.

Response Time (RT)	Estimate	se	t	Pr(>|t|)	
*Intercept (fourth-age)*	7.330	0.021	174.967	0.000	***
Group 60–69 (words + pseudowords)	-0.225	0.020	88.340	0.000	***
Group 70–79 (words + pseudowords)	-0.125	0.020	87.858	0.000	***
Lexicality (words vs. pseudowords)	-0.193	0.016	173.364	0.000	***
Group 60–69: Lexicality (words vs. pseudowords)	0.031	0.010	91.930	0.002	**
Group 70–79: Lexicality (words vs. pseudowords)	0.020	0.009	89.896	0.041	*

*** = p < .001

** = p < .01

* = p < .05

Regarding the accuracy of LDT (Table 4), the generalized linear regression analysis shows that the fourth-age group has a significantly different effect on accuracy compared to the 60–69 age group (β = 0.390, se = 0.167, z = 2.334, p = 0.020), but not to the 70–79 age group (β = 0.242, se = 0.162, z = 1.494, p = 0.135). This indicates that individuals aged 80 years and older make significantly more errors (in recognizing both words and pseudowords) than younger older adults, but not compared to the intermediate group of 70–79 years. Additionally, a main effect is observed among the fourth-age group with lexicality, revealing a higher level of processing facilitation (high accuracy) for words compared to pseudowords (β = 1.041, se = 0.194, z = 5.359, p<0.00).

**Table 4:** Generalized linear mixed-effects regression accuracy results for LDT.

Accuracy	Estimate	se	z	Pr(>|z|)	
*Intercept (fourth-age)*	4.598	0.213	21.568	0.000	***
Group 60–69 (words + pseudowords)	0.390	0.167	2.334	0.020	*
Group 70–79 (words + pseudowords)	0.242	0.162	1.494	0.135	
Lexicality (words vs. pseudowords)	1.041	0.194	5.359	0.000	***
Group 60–69: Lexicality (words vs. pseudowords)	-0.054	0.136	-0.400	0.689	
Group 70–79: Lexicality (words vs. pseudowords)	0.130	0.129	1.003	0.316	

*** = p < .001

** = p < .01

* = p < .05

On the other hand, no reliable interaction effects were found between the third-age groups and lexicality, likely due to the high level of accuracy for both words and pseudowords in those groups (over 92%), and because the differences in accuracy between words and pseudowords were less than 6% in both third-age groups. Nevertheless, post-hoc test analysis (Bonferroni corrected) showed that both third-age groups exhibited a significant difference in the accuracy for words and pseudoword processing (age 60–69, β = 0.675, se = 0.26, z = -2.55, p<0.025; age 70–79, β = 1.179, se = 0.28, z = 3.985, p<0.001; see Fig 2, right panel).

## Discussion

This study aimed to determine the effect of lexicality on advanced aging. The ability to recognize words and pseudowords was explored in people at different stages of aging, and specifically, RT and accuracy performance were compared in a naming task and a LDT between a fourth-age group and two third-age groups. The linear mixed regressions showed a significant increase in the RT needed to recognize both words and pseudowords during the fourth-age in both experiments, indicating that difficulties of lexicality effect appear from the third-age onwards, and this effect is stable and independent of the type of task applied. The fourth-age demonstrated significant lexicality effects in both tasks, where higher recognition costs (higher RT) were observed for pseudowords compared to words. Similarly, the third-age also showed lexicality effects in both tasks (higher RT for pseudowords compared to words), but the observed differences in RT between words and pseudowords were smaller than in the fourth-age, which probably indicates that the lexicality effect increases with age. Regarding overall accuracy, the older fourth-age participants were significantly less accurate in recognizing words and pseudowords compared to their younger peers aged 60–69 years only in LDT. The intermediate group aged 70–79 years showed no significant differences in any of the tasks performed. Similarly, as for accuracy effects for lexicality, the fourth-age group only showed significant differences between words and pseudowords in the LDT, but not in the naming task. Finally, both third-age groups show significant lexicality effects for word versus pseudoword accuracy only in LDT (test post-hoc of Bonferroni), not in the naming task, probably by the ceiling effect produced by the high level of accuracy got in these groups in this task.

The effect of advanced aging on the powerful increase in RT in word and pseudoword recognition in both lexical tasks is justified partially, because elderly people apparently slow down their information processing speed [5, 48]. Specifically, at the neural level, there is evidence on systematic decrease in neural circuitry and reduced neurotransmitter availability being responsible for the reduction in overall cognitive processing speed during old age [49, 50], changes affecting directly the overall ability to recognize words and pseudowords during fourth-age by slowing the weighting between lexical competitors with the input signal.

However, a more concrete explanation to justify the significant increase in RT when recognizing words and specially pseudowords during the fourth-age, corresponds to the progressive decline of fluid intelligence during advanced aging [5, 48]. Several studies have reported that deficits in fluid skills in older adults affect reasoning, problem solving, decision making (required for a lexical decision task in experiment 2), task planning, and mental speed (required for a simple task such as naming in experiment 1). This also includes a decline in skills such as abstract reasoning and the ability to adapt to new situations [4–6, 9]. In this way, the increase in RT in naming experiment and LDT during advanced aging is credited to the fact that fluid intelligence declines abruptly from age 80 onwards [4], which accompanied by the generalized loss of cognitive functionality [51], translates into a substantial decline in information processing speed and the ability to efficiently solve a given task [6, 48, 50].

At the lexical level, we believe that the decline in fluid intelligence during advanced old age could affect word and pseudoword recognition by reducing the speed (RT) of stimulus identification (i.e., quickly relating relevant patterns and features of words/pseudowords), access to word meaning, decision making (responding yes or no to the presented stimulus), and error inhibition such as inappropriately naming a word (experiment 1) or making a wrong decision (experiment 2). In addition, impaired fluid intelligence would reduce the ability to infer the meaning of unknown words from context and contextual cues. Consequently, our results support that the deficit in fluid intelligence during advanced aging seems to generate a powerful effect on the reaction time (RT) required to execute word and pseudoword recognition tasks, imposing cognitive constraints (increased RT) that act independently of the lexical recognition task used (naming vs. LDT).

Regarding the effects of lexicality on RT, the fourth-age group always showed greater processing difficulties in distinguishing pseudowords than words. In turn, the third-age groups also showed lexicality effects in both tasks (characterized by significant difficulties in pseudoword recognition), although these effects were lighter than those observed in the fourth-age group, so it is presumed that the lexicality effect increases as people age. These results confirm that the ability to recognize and read a sequence of letters that has no meaning, and that is ortho-phonologically plausible for Spanish, generates greater processing difficulties than recognizing "real" words throughout the life cycle [17], which according to our results includes very advanced stages of aging. This interference effect generated by pseudowords associated with advanced aging would not be explained, for example, by effects of bilingualism, cognitive performance, educational or another extralinguistic variable. Therefore, it is possible to confirm that the greater cognitive effort associated to the recognize of pseudowords -product of the ineffective search that generates a morphological structure representation valid for the language but nonexistent in the mental lexicon [17, 24, 29]- together with the evident decline in fluid intelligence after the age of 80 [4, 5], lead to a very noticeable lexicality effect during the fourth-age, i.e., a marked cost in the processing of pseudowords over words. Likewise, these results allow us to deduce that the fact that the third-age group also shows difficulties (higher TR) in pseudoword recognition in both LDT (more complex task) and naming task (simpler task) [52–54], would demonstrate that the lexical verification processes performed to ensure that the set of letters presented corresponds or not to a word, would begin to be slightly reduced in early stages of old age independent of the cognitive cost of the task applied.

Similar to what was observed for the RT variable, the overall accuracy for the fourth-age group was significantly lower (both words and pseudowords) compared to their younger peers in the 60–69 age group only in LDT experiment. However, it is striking that the accuracy obtained in the advanced aging group was also very high and close to that shown for the third-age groups (over 96% in naming and over 92% in LDT). It is possible that during advanced aging the high cognitive processing costs have only partially impaired the ability to correctly distinguish (accuracy) both types of stimuli, in contrast to the marked slowing of word and pseudoword recognition. Thus, the accuracy results in the fourth-age appear to be consistent with evidence supporting that the maintenance of crystallized intelligence (vocabulary, knowledge and experiences) during advanced aging would favor the cognitive performance needed to respond to the demands of the environment [16, 22, 23], and would intervene by favoring word recognition accuracy during fourth-age, which would allow counteracting -to some extent- the decline in fluid intelligence and lexical access [4, 55].

Moreover, the comparison of general accuracy between the fourth-age group and the intermediate aging group of 70–79 years did not show significant differences, allowing to hypothesize that the "favoring" role of crystallized intelligence [22, 23] over accuracy of word and pseudoword recognition would be more strongly appreciated in the early stages of old age. It is worth mentioning that although no specific tests of crystallized (and fluid) intelligence were performed in the study sample, the asymmetric behavior of these two types of intelligence is well established during normal aging [4–6, 22, 23], and in general, a "normal asymmetry" is described in which crystallized intelligence could favor performance in certain cognitive and lexical aspects (i.e., maintain high accuracy in experiment 1 and 2), while fluid intelligence could affect others (i.e., increasing the RT in both experiments).

At the lexical level, we believe that the good performance of crystallized intelligence reflected in accumulation of knowledge acquired throughout one’s life, including verbal skills, general knowledge, and accumulated cultural understanding [5–7, 9], could have a significant impact on the accuracy of lexical recognition of words and pseudowords, since broader lexical knowledge generates greater ease of lexical identification (experiment 1), accessing meaning (for words only) and making correct decisions (experiment 2), even those of low lexical frequency. In addition, the experience gained through crystallized intelligence could have provided a broader context and meaning for words, thus facilitating their identification and access to meaning. In sum, with the evidence obtained we assume that individual differences on fluid versus crystallized intelligence asymmetry could be reflected in the magnitude of the lexicality effect showed in both experiments.

On the one hand, the decline in fluid intelligence during the fourth-age could make lexicality a more challenging task for the older person, especially in situations requiring fast processing (such as experiment 1), cognitive flexibility and making a decision (experiment 2), which could increase RT and reduce accuracy during word and pseudoword recognition. In contrast, crystallized intelligence could partially offset the magnitude of the negative effects of decreased fluid intelligence when processing words and pseudowords. A strong lexical knowledge across the lifespan could help older people recognize and understand words more effectively, even in challenging contexts. Thus, the asymmetry between fluid and crystallized intelligence in old age could lead to the adoption of compensatory strategies in lexical processing. For example, older people may rely more on context and contextual cues to infer word meaning and reject pseudowords. They may also use compensatory strategies based on their prior experience and knowledge to overcome possible difficulties in lexical recognition.

In relation to the specific accuracy rate for the lexicality variable, fourth-age people showed a marked lexicality effect, although they only presented meaningful differences between words and pseudowords in the LDT (experiment 2) and not in the naming task (experiment 1), confirming that when the task implied making a decision beyond just naming the stimulus, added to the presence of pseudowords of valid morphological structure for the language, it generated a strong increase in the cognitive load in people aged 80 years and over, which in turn significantly increased the error rate in that group. This situation was also observed in the two third-age groups, although showing significant effects of lexicality in the LTD experiment (test post-hoc of Bonferroni), but not in the naming task, given the ceiling effect that this variable reached in these groups in this experiment. Consequently, it is likely that the benefits associated to crystallized intelligence in the fourth-age [22, 23] may be diminished when the task requires higher cognitive demand (i.e., LDT), and thus, an increased error rate is observed when processing pseudowords. It has been described that during LDT the ability to decide occurs subsequent to signal recognition [37], not only influencing the increase in RT, but also on the total cognitive load of the task [37, 44, 45], and therefore affecting the ability to correctly distinguish pseudowords in advanced aging.

In addition, although the main focus of this research was on the strong cognitive load generated by pseudoword processing during advanced old age, equally the experimental tasks allowed counteracting the behavior of other indexical lexical processes such as lexical identification (determining whether a sequence of letters presented is a word or a pseudoword), semantic access (activating the meaning associated with words and using this information to correctly classify stimuli), decision making (opting whether the sequence of letters is a word or a pseudoword) and inhibition and cognitive control, i.e., rejecting incorrect automatic responses and maintaining focus on the task to avoid errors [17, 19, 24, 36]. These lexical processes were evaluated jointly by means of the variables of RT and accuracy of responses. As a result, we assume that the higher processing costs observed in the fourth-age (reflected by higher RT and lower accuracy) would be indicative of lower overall efficiency in lexical identification, semantic access, decision making and inhibition processes. In this sense, we suppose that LDT not only generated greater cognitive load, but also evidenced greater difficulties in the semantic and lexical evaluation of the stimuli presented, in the time required to respond to the task and the level of accuracy of the responses, with significantly lower results for the advanced aging group.

As for the processing pathways facilitating lexical recognition [27], our results would support that the facilitation effect in word recognition over pseudowords would be explained by the “direct access” associated by the use of the lexical pathway during word processing [19, 28], which allows us to ensure that the simultaneous access to the lexicon and then to the meaning of the word provided by this pathway [19] remains stable even in very advanced stages of old age and would be quite resistant to the changes associated with cognitive decline. On the other hand, Spanish is a language of transparent orthography, that is, the relationship between letters and sounds is direct and consistent [56], therefore, for our study sample it was easier to read pseudowords due to the direct and consistent correspondence between letters and sounds in that language, since the pronunciation rules are more predictable, consistent and can be applied systematically [17]. Although, independent of this property of Spanish (facilitation for pseudowords), the effect of lexicality is consistent throughout the life cycle. Moreover, considering the relative ease of naming pseudowords in Spanish, we believe that these results could be extrapolated to other transparent languages such as Italian or Finnish; but we are not sure if they can be projected to opaque languages where the correspondence between phonemes and writing is neither clear nor predictable (such as English, French or Mandarin Chinese). This means that a straightforward one-to-one correspondence rule between letters and sounds cannot be easily applied to unfamiliar or pseudowords in these languages [29]. Therefore, reading pseudowords in opaque languages entails a higher degree of phonological processing and a greater cognitive load compared to reading real words [17, 29]. It requires additional effort to correctly identify and pronounce pseudowords, as readers cannot rely on the letter-sound associations they have previously learned [17]. So it would be interesting to investigate the effect of lexicality on advanced aging in native speakers of these languages.

Another important point to note is the behavior of "no" responses in the LDT (experiment 2). In general, there is evidence that "no" responses to pseudowords have a higher RT than "yes" responses to words [24, 37, 57, 58]. In the case of older adults, this higher RT for "no" responses could be due to two reasons: 1) as a result of the lexical-orthographic verification process that pseudowords require and that allows them to be rejected (lexicality effect), and therefore the “no” response is generated after a certain amount of processing time has elapsed (the pseudoword deadline is flexible and is extended when the system detects more global lexical activity early in processing [27]); or 2) as a consequence of the slower speed of information processing in old age [5, 6] (i.e., fluid intelligence decline). However, with respect to the latter, and considering that cognitive slowing is generalized, several LDT studies comparing RT in young and older adults have shown that both types of responses (yes/no) increase in latency with age [44, 45]. Therefore, we believe that the "no" responses obtained in experiment 2 are mainly due to lexicality effects (words vs. pseudowords) rather than other age-related effects. The "no" responses resulting from the lexicality effect are supported by Perea et al. [24], who, like our study, used plausible pseudowords for Spanish based on high and low lexical frequency words. As a result, it was shown that the "no" response to the pseudoword really corresponded to the verification performed at the lexical-orthographic level, and its latencies (RT) could vary according to the properties of the processed pseudoword (variable deadline mechanism).

## Conclusion

The study provides a better understanding of the lexicality effect in advanced stages of aging. Pseudoword processing seems to resist well the cognitive decline typical of aging, since it begins to manifest features of marked deterioration only after the age of 80. However, lexicality difficulties may begin slightly in the early stages of aging. The naming and LDT tasks showed that the fourth-age group displays a patent increase in RT when distinguishing both words and pseudowords compared to both third-age groups and shows a lower overall accuracy rate. Regarding the specific effects of lexicality on RT, the fourth-age group always showed greater processing difficulties in distinguishing pseudowords than words. In turn, the third-age groups also showed lexicality effects in both tasks, although these effects were lighter than those observed in the fourth-age group, so it is presumed that the lexicality effect increases as people age.

The decline in fluid intelligence during advanced old age could affect word and pseudoword recognition by reducing the speed of stimulus identification, access to word meaning, decision making, and error inhibition. In addition, we also supported that the maintenance of crystallized intelligence reinforces the accuracy of lexical recognition throughout old age, although this effect would be stronger in the early stages of old age (60–69 years) and to a lesser extent in very advanced stages (80+). Finally, as for the processing pathways facilitating lexical recognition, our results support that the facilitation effect in word recognition over pseudowords would be explained by the “direct access” associated by the use of the lexical pathway during word processing, which allows us to ensure that the simultaneous access to the lexicon and then to the meaning of the word provided by this pathway remains stable even in very advanced stages of old age and would be quite resistant to the changes associated with cognitive decline. Furthermore, it would also support the idea that general comprehension remains stable during advanced aging.

## Limitations and future directions

The results found should be considered as basic empirical evidence on the effects of lexicality and the role of pseudowords in advanced aging. Future studies should explore the physiological correlation of the lexical behavior described in this investigation, expanding the knowledge of how people aged 80 years and older manifest their linguistic and cognitive changes when processing words and pseudowords. Finally, a limitation of this research would be the fact that only the lexicality effect through visual recognition was studied, which leaves the question of whether these findings would transfer to other modalities, such as auditory recognition.

## Supporting information

S1 File List of trials by experimental task. (PDF)

S2 File Results of complementary analysis for experiment 1 and experiment 2. (PDF)
