# Supplementary material for: Word or pseudoword? The lexicality effect in naming and lexical decision tasks during advanced aging
Source: PLoS One. 2024 Feb 29;19(2):e0299266. doi: 10.1371/journal.pone.0299266 (PMC10903840; doi:10.1371/journal.pone.0299266)
Supplement: S1 File — (PDF) [file pone.0299266.s001.pdf]

S1. List of trials by experimental task

1. Naming experiment: List of experimental and filler trials.

Words (2-3 syllable)	Condition Trial	Lexical frequency (1=High, 0=Low)	PSF (1=High, 0=Low)	Pseudowords
Comer	Experimental	1	1	Coler
Abierto	Experimental	1	1	Abierco
Palabras	Experimental	1	1	Palafras
Local	Experimental	1	1	Lotal
Tomar	Experimental	1	1	Tolar
Militar	Experimental	1	1	Milicar
Médico	Experimental	1	1	Médito
Música	Experimental	1	1	Músina
Colegio	Experimental	1	1	Colemio
Cabeza	Experimental	1	1	Cabeta
Casa	Experimental	1	1	Cafa
Ministro	Experimental	1	1	Minisclo
Problema	Experimental	1	1	Probleja
Saber	Experimental	1	1	Sater
Animal	Experimental	1	1	Anital
Tribu	Experimental	1	0	Tricu
Bravo	Experimental	1	0	Bramo
Frases	Experimental	1	0	Frates
Bronce	Experimental	1	0	Bronte
Montaña	Experimental	1	0	Montaca
Jardines	Experimental	1	0	Jardifes
Barco	Experimental	1	0	Olvinar
Olvidar	Experimental	1	0	Olvinar
Castigo	Experimental	1	0	Castino
Cría	Experimental	1	0	Bría
Bruto	Experimental	1	0	Bruyo
Trono	Experimental	1	0	Troyo
Crudo	Experimental	1	0	Cruno
Signo	Experimental	1	0	Sigco
Tregua	Experimental	1	0	Trecua
Calar	Experimental	0	1	Canar
Nobel	Experimental	0	1	Nótel
Empuñar	Experimental	0	1	Empugar
Infectar	Experimental	0	1	Infecnar
Distal	Experimental	0	1	Disnal

Pajar	Experimental	0	1	Panar
Dilatar	Experimental	0	1	Dilanar
Macaco	Experimental	0	1	Macato
Encías	Experimental	0	1	Enmías
Codorniz	Experimental	0	1	Codorfís
Mucosas	Experimental	0	1	Mucotas
Rebatir	Experimental	0	1	Rebanir
Tifus	Experimental	0	1	Tillus
Profesar	Experimental	0	1	Profetar
Sicario	Experimental	0	1	Sicanio
Palpar	Experimental	0	0	Palnar
Polvorín	Experimental	0	0	Polvomín
Baldío	Experimental	0	0	Balmío
Croquis	Experimental	0	0	Cromis
Clonar	Experimental	0	0	Clocar
Garfio	Experimental	0	0	Garmio
Factibles	Experimental	0	0	Factiples
Brotado	Experimental	0	0	Brotafo
Captan	Experimental	0	0	Capcan
Balsero	Experimental	0	0	Balseno
Yerra	Experimental	0	0	Yela
Rencillas	Experimental	0	0	Rencidas
Bujías	Experimental	0	0	Bulías
Múltiplos	Experimental	0	0	Múltinos
Bastidor	Experimental	0	0	Bastinor
Rodillo	Filler	-	-	Rodino
Meses	Filler	-	-	Meles
Razón	Filler	-	-	Racón
Abedul	Filler	-	-	Abenul
Color	Filler	-	-	Cotor
Medio	Filler	-	-	Mefio
Rebatir	Filler	-	-	Rebanir
Burdel	Filler	-	-	Burnel
Carroña	Filler	-	-	Carroca
Bloque	Filler	-	-	Blote
Croacia	Filler	-	-	Croania
Frutas	Filler	-	-	Frumas
Franela	Filler	-	-	Franena
Brillante	Filler	-	-	Brillanque
Cloaca	Filler	-	-	Cloana

2. **Lexical decision task:** List of experimental and filler trials.

Words (2-3 syllable)	Condition Trial	Lexical frequency (1=High, 0=Low)	Imaginability (1=High, 0=Low)	Pseudowords
Agua	Experimental	1	1	Atua
Niña	Experimental	1	1	Niya
Manos	Experimental	1	1	Mabos
Cielo	Experimental	1	1	Cieno
Comida	Experimental	1	1	Comifa
Corazón	Experimental	1	1	Coralón
Cuerpo	Experimental	1	1	Cuerlo
Hija	Experimental	1	1	Hina
Libro	Experimental	1	1	Licro
Luna	Experimental	1	1	Luva
Madera	Experimental	1	1	Madeya
Mujer	Experimental	1	1	Muler
Parque	Experimental	1	1	Parne
Médico	Experimental	1	1	Médino
Verano	Experimental	1	1	Veraco
Capaz	Experimental	1	0	Carraz
Intención	Experimental	1	0	Intenfión
Valores	Experimental	1	0	Valoyes
Recurso	Experimental	1	0	Recurno
Razón	Experimental	1	0	Ranón
Real	Experimental	1	0	Rean
Voluntad	Experimental	1	0	Voluncad
Cierto	Experimental	1	0	Cierno
Sensación	Experimental	1	0	Senfación
Carácter	Experimental	1	0	Caráccer
Conciencia	Experimental	1	0	Concientia
Causa	Experimental	1	0	Cauna
Lógica	Experimental	1	0	Lógiba
Percepción	Experimental	1	0	Pernepción
Moral	Experimental	1	0	Monal
Bizcocho	Experimental	0	1	Monal
Zorzal	Experimental	0	1	Zornal
Almeja	Experimental	0	1	Almena
Maceta	Experimental	0	1	Macena
Cubeta	Experimental	0	1	Cubena
Biombo	Experimental	0	1	Biomfo

Molusco	Experimental	0	1	Molusno
Simio	Experimental	0	1	Sinio
Brasa	Experimental	0	1	Brata
Madeja	Experimental	0	1	Madela
Equino	Experimental	0	1	Equimo
Bisturí	Experimental	0	1	Bustuní
Joroba	Experimental	0	1	Jorota
Betún	Experimental	0	1	Belún
Morral	Experimental	0	1	Monal
Cuantiosa	Experimental	0	0	Equimo
Conciso	Experimental	0	0	Consino
Alarde	Experimental	0	0	Alarne
Morboso	Experimental	0	0	Morbono
Certera	Experimental	0	0	Certena
Indaga	Experimental	0	0	Indana
Agobio	Experimental	0	0	Agonio
Abruma	Experimental	0	0	Abruya
Sadismo	Experimental	0	0	Sadisto
Pérfido	Experimental	0	0	Pérfiyo
Melosa	Experimental	0	0	Melota
Burdo	Experimental	0	0	Burco
Fascina	Experimental	0	0	Fascita
Tortuoso	Experimental	0	0	Tortuono
Sórdido	Experimental	0	0	Sórdifo
Usurpar	Filler	-	-	Usurlar
Esternón	Filler	-	-	Esterfón
Dormir	Filler	-	-	Dorbir
Farsante	Filler	-	-	Farsanbe
Humor	Filler	-	-	Hutor
Lengua	Filler	-	-	Lenfua
Comulgar	Filler	-	-	Comultar
Pomposo	Filler	-	-	Pompono
Deseo	Filler	-	-	Deteo exisne
Fuego	Filler	-	-	Fueno
Especial	Filler	-	-	Espefial
Ampolla	Filler	-	-	Ampota
Frotar	Filler	-	-	Fronar
Diablo	Filler	-	-	Diaclo
Existe	Filler	-	-	Exisne
