# Supplementary material for: Word or pseudoword? The lexicality effect in naming and lexical decision tasks during advanced aging
Source: PLoS One. 2024 Feb 29;19(2):e0299266. doi: 10.1371/journal.pone.0299266 (PMC10903840; doi:10.1371/journal.pone.0299266)
Supplement: S2 File — (PDF) [file pone.0299266.s002.pdf]

S2. Results of complementary analysis for Experiment 1 and Experiment 2 (with age as a continuous predictor)

Experiment 1: Response Time.

The linear model used was: lmer(logRT ~ Age * Lex + (1 + Lex | Participant) + (1 + Age | Item)

Table A1. Linear mixed-effects regression response time results for naming experiment with age as a continuous predictor.

	Estimate	*se*	*t*	*p*	
(Intercept)	6,940	0,023	307,461	0,000	***
Age	0,136	0,021	6,637	0,000	***
Lexicality	-0,074	0,010	-7,241	0,000	***
Age * Lexicality	-0,007	0,004	-1,951	0,054	.

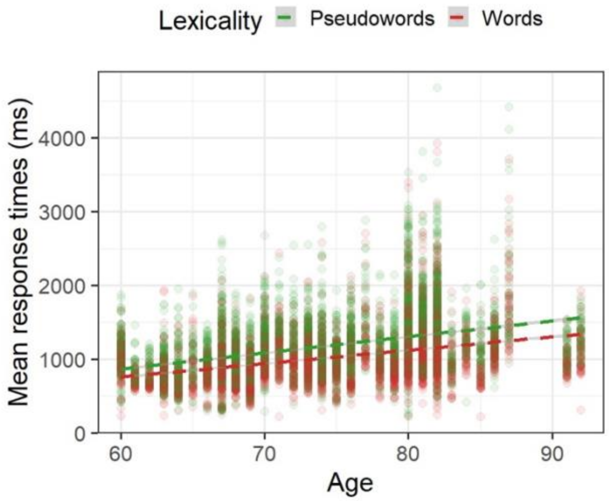

Figure A1. Mean RT as a function of age (as a continuous predictor) and lexicality (words vs. pseudowords) in the naming task for Experiment 1.

Linear regression analysis on participants' RT shows a significant effect of age (as a continuous variable) on RT (β= 0.136, se= 0.021, t= 6.637, p<0.00). In this regard, figure A1 corroborates that the RT required to recognize words and pseudowords increases with age. However, it is not possible to establish at what point in old age the greatest difficulties begin to manifest themselves. In addition, a significant effect of lexicality on RT is observed (β= -0.074, se= 0.010, t= -7.241, p<0.00), showing a better performance of words (<RT) versus pseudowords throughout old age. Finally, no significant interaction effect between age and lexicality on RT is observed.

Experiment 1: Accuracy.

The generalized linear model used was: glmer(acc ~ Age * Lex + (1 + Lex | Participant) + (1 + Age | Item)

Table A2. Generalized linear mixed-effects regression accuracy results for naming experiment with age as a continuous predictor.

	Estimate	*se*	*z*	*p*	
(Intercept)	11,695	1,335	8,759	0,000	***
Age	-1,996	1,148	-1,739	0,082	.
Lexicality	0,239	0,721	0,332	0,740	
Age * Lexicality	0,242	0,636	0,380	0,704	

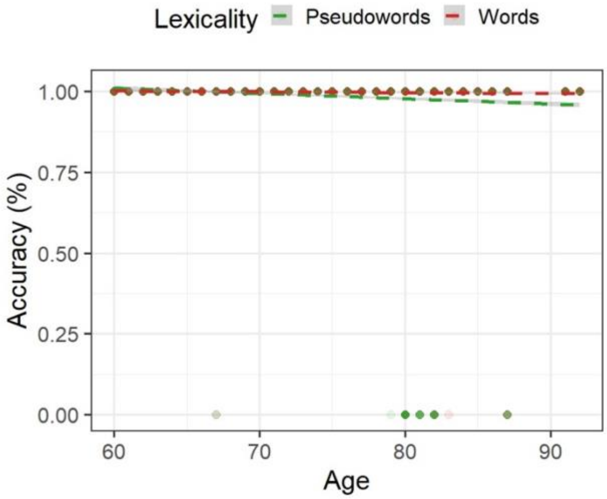

Figure A2. Accuracy percentage as a function of age (as a continuous predictor) and lexicality (words vs. pseudowords) in the naming task for Experiment 1.

For the naming experiment, generalized linear regression shows no significant effect of age (aging), lexicality (word vs. pseudowords) and the interaction between age and lexicality on the accuracy variable (see figure A2).

Experiment 2: Response Time.

The linear model used was: lmer(logRT ~ Age * Lex + (1 + Lex | Participant) + (1 + Age | Item)

Table A3. Linear mixed-effects regression response time results for LDT experiment with age as a continuous predictor.

	Estimate	*se*	*t*	*p*	
(Intercept)	7,331	0,023	317,012	0,000	***
Age	0,176	0,019	9,298	0,000	***
Lexicality	-0,193	0,016	-12,334	0,000	***
Age * Lexicality	-0,022	0,008	-2,693	0,008	**

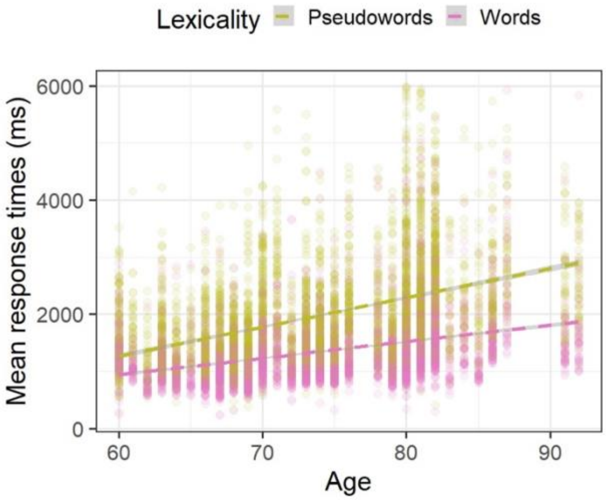

Figure A3. Mean RT as a function of age (as a continuous predictor) and lexicality (words vs. pseudowords) in the LDT for Experiment 2.

Linear regression analysis shows a significant effect of age on RT (β= 0.176, se= 0.019, t= 9.298, p<0.00). In this sense, figure A3 shows that as age increases so does the TR required to recognize words and pseudowords; however, this analysis also does not allow us to establish at what point in old age the greatest difficulties begin to manifest themselves (similar to naming experiment). In addition, a significant effect of lexicality on RT is observed (β= -0.193, se= 0.016, t= -12.334, p<0.00), which shows better RT performance for words over pseudowords throughout old age. Finally, a significant effect of age and lexicality over RT is observed (β= -0.022, se= 0.008, t= -2.693, p<0.008). This interaction reflects an increase in the difference between RT for words and pseudowords with an increase in age (see figure A3).

Experiment 2: Accuracy.

The generalized linear model used was: glmer(acc ~ Age * Lex + (1 + Lex | Participant) + (1 + Age | Item)

Table A4. Generalized linear mixed-effects regression accuracy results for LDT experiment with age as a continuous predictor.

	Estimate	*se*	*z*	*p*	
(Intercept)	4,554	0,177	25,733	0,000	***
Age	-0,269	0,121	-2,218	0,027	*
Lexicality	1,033	0,171	6,057	0,000	***
Age * Lexicality	0,007	0,112	0,061	0,952	

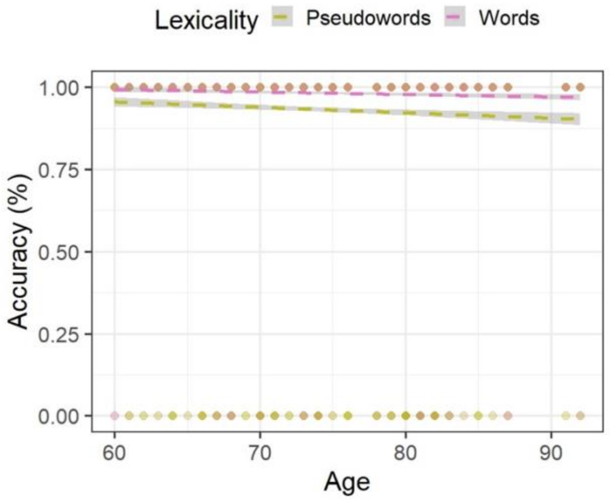

Figure A4. Accuracy percentage as a function of age (as a continuous predictor) and lexicality (words vs. pseudowords) in the LDT for Experiment 2.

Generalized regression analysis shows a significant effect of the age factor on the accuracy variable (β= -0.269, se= 0.121, z= -2.218, p<0.027). This result reflects a reduction of accuracy response with increasing age. In addition, there is a significant effect of lexicality on accuracy (β= 1.033, se= 0.171, z= 6.057, p<0.000), where words show a better accuracy rate compared to pseudowords throughout old age (see figure A4). Finally, there is no significant effect of the interaction between age and lexicality on accuracy.

In sum, it is possible to see that these new models do not provide much different information than the models proposed in the manuscript (where we divided the sample into groups). Moreover, the new models do not allow us to determine the exact age at which the greatest difficulties in word and pseudoword recognition are observed, given that the relations observed (between age and response times and accuracy) are quite linear.
